# Characteristics and Quality of Flame-Retarded Ramie Fabrics for the Development of Functional Textiles

**DOI:** 10.3390/ma17061416

**Published:** 2024-03-20

**Authors:** Asri Peni Wulandari, Erlin Karlina, Eric Tanudjaja, Abdul Rohmat, Joko Kusmoro, Muhammad Fadhlillah, Karlina Somantri, Roni Sahroni, Widya Fatriasari

**Affiliations:** 1 Department of Biology, Faculty of Mathematics and Natural Science, Padjadjaran University, Sumedang 45363, Indonesia; joko.kusmoro@unpad.ac.id; 2 Center for Study of Bioprospection of Natural Fiber and Bioresources, Faculty of Mathematics and Natural Science, Padjadjaran University, Bandung 40132, Indonesia; 3 PT Dymatic Chemicals Indonesia, Bandung 40552, Indonesia; erlinkarlina512@gmail.com (E.K.); eric.tanudjaja@dymatic.co.id (E.T.); 4 PT Haramai Jaya Nusantara, Bandung 40132, Indonesia; abdul.rohmat@rajantara.com; 5 Department of Chemistry, Faculty of Mathematics and Natural Science, Padjadjaran University, Sumedang 45363, Indonesia; fadhlillah@unpad.ac.id; 6 Bandung Polytechnic of Textile Technology, Bandung 40272, Indonesia; karlina.somantri@gmail.com (K.S.); roni.sahroni@stttekstil.ac.id (R.S.); 7 Research Center for Biomass and Bioproduct, National Research and Innovation Agency, Bogor 16911, Indonesia; widy003@brin.go.id

**Keywords:** natural fiber, ramie fabric, flammability, flame retardant, coating, functional textile

## Abstract

Cellulose fabric testing for flame-retardant studies is frequently necessary in various textile applications. Natural cellulose material from ramie (*Boehmeria nivea*) is being promoted as an alternative raw material for the development of fire-resistant fabrics. This research aims to optimize the coating process of ramie fabric using a phosphorus-based flame retardant (FR) to enhance its flame-retardant characteristics. The FR treatment involves bleaching the fabric with H_2_O_2_; followed by fabric finishing using a formula comprising 3% (*v*/*v*) hydroxymethyl resin; phosphoric acid (2%); and two formulations of the flammable agent Flamatic DM-3072N: 40% (*v*/*v*) and 50% (*v*/*v*), applied using the pad-dry-cure method. The flame-retardant properties of the treated fabric are evaluated through flammability testing based on the ASTM D6413-08 standard, limiting oxygen index (LOI) analysis, and micrograph surface structure analysis with SEM. The results indicate that ramie fabric treated with the FR-50% material exhibits superior fire resistance, preventing fire spread on the fabric with a char length of 15–30 mm and a LOI value of 29. These findings highlight the potential of FR-treated ramie fabrics for various industries, including the automotive and protective clothing industries.

## 1. Introduction

Wear performance represents one of the most rapidly expanding segments within the textile industry. Market expansion is fueled by the introduction of novel advancements in fabric technology. The evolution of the fabric sector has spurred the creation of high-tech protective textiles characterized by enhanced tensile strength, cut resistance, abrasion resistance, and durability.

Natural fibers such as cotton and linen are frequently employed as raw materials in textile manufacturing [1,2], owing to their extensive presence in the market due to their remarkable attributes of softness, comfort, and hydrophobicity [3,4].

The utilization of natural fibers in various applications is still hindered by their mechanical quality, particularly their low strength compared to synthetic fibers [5]. This inherent weakness in natural fibers stems from their polar nature and abundant hydrogen bonds, rendering them susceptible to water absorption, particularly in high-humidity (hydrophilic) environments. The absorption of water disrupts molecular chain interactions, leading to heightened water diffusivity and potential uptake of water vapor from the atmosphere [6]. The ingress of water into fibers can lead to weathering, diminished compatibility of fiber composites, an increase in weight, and susceptibility to degradation [7].

Despite these disadvantages, natural fibers or cellulose fibers also possess several advantages. For example, cotton fiber and pineapple fiber are non-flammable materials with an LOI value of approximately 18% [8,9,10]. Several methods have been explored to enhance the resistance of cotton-based textiles, including the sol-gel technique [11,12], layer-by-layer (LbL) assembly [13,14], plasma processing [15,16], flame-retardant finishing technology [17,18], coating technology [19], and surface-grafting treatment [20].

Renewable resources, such as DNA [21], chitosan [22,23], protein [24], starch [15,25], and phytic acid (PA) [16,22,26,27] have been employed as flame retardants over the past decade. The results demonstrate a significant impact of the mentioned renewable resources in enhancing the fire resistance of natural fiber-based fabrics. PA, easily extractable from various plant parts except leaves [28], contains six phosphate groups and twelve hydroxyl groups, enabling interaction with almost all types of reactive groups [22].

Dittenber (2012) [29] reported that ramie fiber, a cellulose-based natural fiber, exhibits the highest mechanical properties among other natural fibers. In our previous research, the modification of ramie fibers through cross-linking methods by adding CA (citric acid) and SHP (Sodium Hypophosphite) was found to enhance the mechanical strength of ramie fibers by up to 20 times [30]. This highlights the potential of enhancing ramie fibers through refinement methods for various applications. In this study, a highly strengthened ramie fabric will be developed for fire-resistant functional applications.

Several methods reported for enhancing the fire resistance properties of modified ramie fabric have utilized the Schiff base reaction between aldehyde groups and amino groups to pretreat the fabric surface. In this regard, the utilization of Polyethyleneimine (PEI) acts as the positive charge, while ammonium polyphosphate (APP) serves as the negative charge [31]. Another method involves using a polyethylenimine (PEI) solution and phosphoric acid zirconium [32]. Additionally, a method using an adhesive layer of rosin benzoxazine containing a catalyst mixture in the form of methyl tosylate and iron acetylacetonate with the fire inhibitors being polyethyleneimine (PEI) and poly bis phenolic phenyl phosphate ester [33]. Although these methods significantly improve fire resistance, the extensive use of finishing additives and organic solvents still limits their application prospects. These methods are conducted with multilayer module units assembled layer by layer, requiring the assembly of 15–30 bilayers to achieve satisfactory fire-resistant modifications, thus constraining their widespread use in the industry.

In this research, further investigation was conducted on ramie fabric as a cellulose material, dissolving it using a simpler and more practical method without layering lamination processes. Additionally, testing was conducted using a non-ionic and non-toxic substance, Flamatic DM-3072N, as a nitrogen derivative product containing organophosphorus compounds. The flame-retardant capability will be assessed through characterization of ramie fabric and quality flammability tests.

## 2. Materials and Methods

### 2.1. Materials

The fine-fiber woven ramie fabric (KR-T01) is a specially woven fabric made from ramie fiber, produced through the spinning and fabric provision processes conducted by PT Haramai Jaya Nusantara, Bandung, Indonesia. The characteristics of the plain fabric are shown in Table 1. The chemicals and flame retardant (FR) DM-3072N (a nitrogen derivate containing an organophosphorus compound) were provided by PT Dymatic Chemicals Indonesia, Bandung, Indonesia. FR DM-3072N was chosen due to its suitability for durable flame-retardant finishing of cotton fabric, providing the fabric with excellent flame-retardant effect, non-toxicity, and good compatibility [34]. 

### 2.2. Characterization of Ramie Fabric

Ramie fabric samples, before being treated with FR, were characterized based on the SNI 0458:2013 standard: SNI ISO 7211-1:2010 for fabric construction/woven types; the thread number test with SNI ISO 7211-5:2010, the thread total test (SNI ISO 7211-2:2010); the fabric thickness test (SNI ISO 5084:2010), the fabric weight test (SNI ISO 3801:2010), fabric width (SNI ISO 22198:2010), the size change test after washing (SNI ISO 6330:2015), fabric tensile strength/2.5 cm (SNI 0276:2009), as well as fabric tear strength testing (SNI 0521:2008).

#### 2.2.1. Peroxide Treatment

The bleaching process was conducted according to Repon et al. (2021) [35] with some modifications. This process aims to eliminate lignin and other impurities from the ramie fabric, resulting in the loss of certain cellulose fibers along with lignin content [36]. In brief, the treatment with H_2_O_2_ [Merck] was carried out using the following recipe: hydrogen peroxide at a concentration of 4% (*w*/*v*), Sodium hydroxide at 2% (*w*/*v*) [Sigma-Aldrich, Merck KGaA, Darmstadt, Germany], and a stabilizing agent at 0.1% (*w*/*v*) [DIMASTAB CEB], with a material to liquor ratio of 1:40. The process was conducted at 100 °C for 30 min. Subsequently, after bleaching, the fabric underwent a cold wash with aquades at room temperature for 10 min followed by drying at 120 °C for 3 min.

#### 2.2.2. Preparation of Flame-Retardant Fabric

The process of finishing the ramie fabric with a flame retardant refers to the Raymond Li (2015) method [37] with modifications. The ramie fabric samples were coated with basal recipes based on 3% hydroxyl methyl resin [DYMAFIN DM-3511A] and 2% phosphoric acid with variation of Flamatic DM-3072N at concentrations of 40% (*v*/*v*) and 50% (*v*/*v*). These concentrations were selected based on their superior flame-retardant properties compared to other variations tested previously. The treatment process utilized the pad-dry-cure method at temperatures ranging from 150 to 170 °C for 1–5 min. The FR process was evaluated using the standard test method for the flame resistances of textiles (the vertical test) [Method: ASTM D6432].

### 2.3. Flammability Testing and Evaluation

The combustion resistance of the treated specimens was evaluated using the vertical test method, specifically ASTM D6413. The specimens used for this test were sized 10 × 3 cm. Vertical testing was carried out to evaluate the flame spread characteristics on the textile specimens. A flame source was ignited beneath the specimen for a duration of 10 s and then removed, allowing the material to self-extinguish. The evaluation of combustion was performed according to predefined criteria outlined in Table 2.

The flammability assessment aimed to evaluate the fire resistance of the fabric samples treated with FR formulations intended for potential development as a functional textile. During this stage, the burning time and char length were examined. Cheng et al. (2016) [38] categorized fabrics into three groups based on the vertical burning test according to GB 8624-2012.

### 2.4. Limiting Oxygen Index (LOI)

The limiting oxygen index (LOI) represents the minimum oxygen fraction required in a mixed environment of oxygen (O_2_) and nitrogen (N_2_) to sustain vertical combustion. The test was conducted using a Dynisco Plastics LOI analyzer, following ASTM Standard Method D2863 [39]. The sample dimensions were 52 mm × 140 mm. The sample was ignited from the top end using a butane flame (within 20 s) and allowed to burn downwards. The test was repeated by adjusting the volumetric flow rate of O_2_ and N_2_ until a specific burning time of 3 min or a burning length of 50 mm was achieved. LOI is calculated as follows:

$$LOI\left(\%\right)=\frac{\left[O_{2}\right]}{{[O}_{2}]+{[N}_{2}]}\times 100$$

where [O_2_] is the volumetric discharge of oxygen and [N_2_] is the volumetric discharge of nitrogen.

### 2.5. Fourier-Transform Infrared (FTIR) Analysis

Fourier-transform infrared (FTIR) analysis was conducted on the ramie fabric samples with FR coating treatment and without FR coating treatment. The analysis was performed using a Perkin Elmer FTIR (Model: System 2000, PerkinElmer Corporation, Waltham, MA, USA) employing the ATR (attenuated total reflectance) method with spectrum two specs, resolution 4 cm^−1^, and 16 scans. This FTIR analysis generated data in the form of absorbance or transmittance graphs. The FTIR analysis was conducted quantitatively by observing the spectrum’s shape at specific peaks and the intensity of the FTIR graph. The tests were carried out in the wave number range 400–4000 cm^−1^, with a resolution set at 2 cm^−1^ to examine the absorption peak of the OH group (hydroxyl) at the wave number 3000–3650 cm^−1^ and the absorption of -COOR′ (ester) at the wave number 1710–1780 cm^−1^.

### 2.6. NMR Analysis

Nuclear magnetic resonance (NMR) analysis was used to obtain information about the structure and conformation of chemical compounds. NMR spectroscopy serves as a reliable method for elucidating the structure of organic compounds [40]. The NMR analysis was conducted utilizing a Bruker AV III 600 spectrometer (Billerica, MA, USA). Signals obtained from NMR spectroscopy provide information about interactions between nuclei and electrons as well as nuclear interactions, aiding in the determination of chemical compound structures [41]. The resulting NMR spectrum comprises one or more resonance peaks at specific frequencies.

### 2.7. Micrograph Surface Structure Analysis

Micrograph surface structure analysis was conducted to observe the morphology of ramie fabric samples with and without FR coating, carried out with an acceleration voltage of 10 kV. The surface structure of the ramie fabric was observed using a scanning electron microscope (SEM) equipped with a FE-SEM Thermo Scientific Quattro S completed with an EDS detector, WetSTEM, heating stage, and tensile stage. The samples were coated with gold using a Cressington 208HR High-Resolution sputter coater (Cressington Scientific Instruments, Watford, UK). Magnification ranged from 100 to 10,000 times until surface objects were visible.

## 3. Results and Discussions

### 3.1. The Structural Properties of the Fabrics

The ramie fabric utilized in this study is a woven material identified by the code KR-T01. The property characterization findings of the ramie fabric, determined through testing according to the Indonesian National Standard (SNI), are presented in Table 1.

### 3.2. Flame Retardancy and Flammability

**Figure 1:**
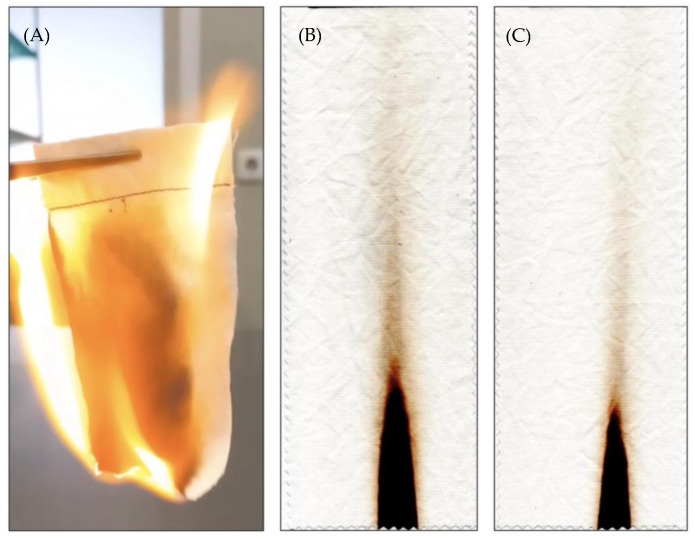
Vertical flammability test of: (**A**) the untreated ramie fabric (**B**) 40%—FR (**C**) 50%—FR.

To assess the fire-resistant properties of the treated ramie fabric, we conducted limiting oxygen index (LOI) and vertical flammability test on ramie fabrics treated with various concentrations of the Flamatic retardant. The results are presented in Figure 1 and Table 3. As shown in Figure 1, the untreated ramie fabric exhibited no fire resistance during the vertical flammability test, evidenced by a flame time of 20 s and a glow time of 78 s.

Additionally, when the concentration of the Flamatic retardant is set to 40% and 50%, the flame time and glow time for all treated ramie fabrics are reduced to 0 s, while the char length ranges from 15 to 30 mm. This indicates that the treated ramie fabrics successfully passed the vertical flammability test (Table 3). Various natural materials, including cotton and wool, treated with different FR methods and materials, have exhibited signs of improved fire resistance. The findings of this study suggest that ramie fabric treated with Flamatic-DM-3072N demonstrates superior quality as a fire-resistant functional textile.

The phosphorus compounds contained in the flame retardant act as an acid donor or dehydrogenation catalyst. Meanwhile, the nitrogen compounds act as blowing agents, releasing the necessary gas (N_2_) for foam formation [42]. The Phosphorus–Nitrogen synergistic flame retardant was more widely employed due to its excellent efficiency to achieve optimal performance of FR [43,44].

Moreover, these compounds have broader applications and are currently gaining increased attention. The flame-retardant mechanism of the traditional phosphorus flame retardant is to form phosphoric acid to promote cationic crosslinking after combustion. The addition of nitrogen leads to the emission of substantial amounts of non-toxic, non-combustible gases when heated. This synergistic effect of nitrogen and phosphorus contributes significantly to achieving the desired flame-retardant properties [45,46].

**Table 3:** Vertical flammability test of ramie fabric and compared to other natural fiber material.

No.	Material	After Flame Times (s)	After Glow Times (s)	Dripping	Ignition	Char Length (mm)	Reference
1.	Ramie fabric (untreated)	20	78	No	No	-	this work
2.	Ramie fabric 40%—FR	0	0	No	No	30	this work
3.	Ramie fabric 50%—FR	0	0	No	No	15	this work
4.	Cotton fabric (ASMPEA treated)	0	0	No	No	48–64	[47]
5.	Cotton fabric (untreated)	6.9	44.6	-	-	-	[48]
6.	Cotton fabric (350 g/L-SPPTMS)	0	0	-	-	81	[48]
7.	Wool fabric (PA treated)	5–15	15–30	-	-	150–200	[49]

According to the classification of FR fabric based on the vertical burning test from GB 8624-2012 [38], the ramie fabric which treated with the Flamatic retardant at 40–50% could be classified with a B1 classification: char length ≤ 15 cm, after flame time ≤ 5 s, after glow time ≤ 15 s.

### 3.3. Tensile Characterization

The tensile strength test was only conducted on KR-T01—50% FR due to its lower char length and having better optimization results compared to KR-T01—40% FR. In addition, the KR-T01—40% FR sample was only used to assess its flame-retardant characteristics. The characterization of ramie fabric based on tensile strength revealed changes in the fabric’s tensile strength before and after treatment (Table 4). The results showed that the tensile strength of KR-T01—50% FR was reduced by more than 10 MPa (40.60 ± 3.32) compared to the KR-T01—Non FR (untreated) (56.69 ± 5.84). This indicates that the mechanical properties of ramie fabric treated with 50% FR were weakened due to the flame-retardant finishing.

### 3.4. Limiting Oxygen Index (LOI)

The method widely used to assess the combustion ability or flammability of polymers is the LOI. Generally, materials with an oxygen index < 22 are classified as flammable, those with an oxygen index between 22 and 27 are considered combustible, and those with an oxygen index > 27 are considered refractory [50]. Below is a comparison of LOI data between fabrics crafted from ramie fiber and fabrics made from other fibers or commercial fabrics (Table 5).

The LOI value of untreated ramie fabric is 22%, an indication of its combustible properties. Conversely, the results of the LOI analysis of fabrics treated with FR (KR-T01—50% FR) demonstrate that fire resistance can be enhanced with a flame-retarding agent, yielding an LOI of 29%. This places the fabric in the category of refractory properties, comparable to cotton material treated with SPPTMS [48]. These results showed the advantages of using ramie material for the production of functional textiles with fire-resistant and heat-resistant characteristics.

Woven ramie fabric treated with a flame-retardant coating with the formula Flamatic DM-3072N showed potential as a raw material for producing functional textiles with flame retardant properties. Possible products derived from this flame-retardant ramie fabric include flame-retardant clothing, curtains, wall coverings, interior fabric-based products, and more.

### 3.5. Structural Reaction Analysis

#### 3.5.1. FTIR Spectra

**Figure 2:**
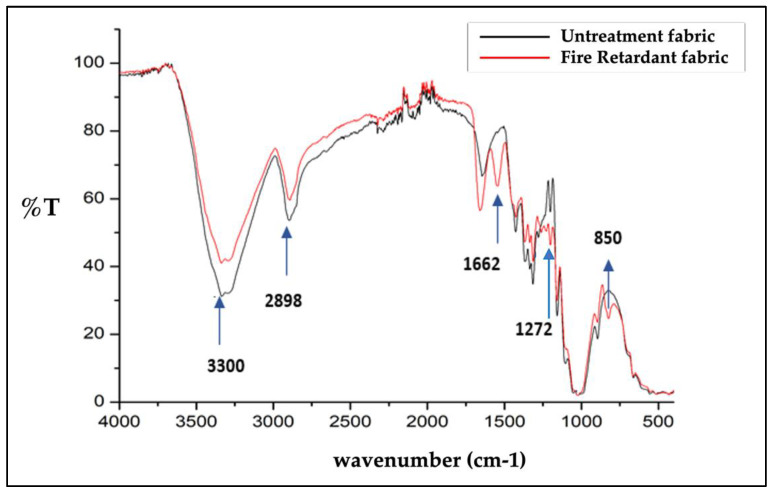
FTIR spectra of ramie fabric untreated and treated with FR formula.

In Figure 2, the FTIR spectra of ramie fabric 50%—FR are presented. The near-infrared absorption signal at 3335 cm^−1^ corresponds to the oscillation of the NH_4_^+^ group [53], while at 2898 cm^−1^ shows CH and CH_2_ stretching [54]. Peak absorption caused by the presence of P–O, C–N, and N–H groups can be seen at 1287.6 cm^−1^, 1406.8 cm^−1^, and 1650.0 cm^−1^ [55,56]. The signal intensity of the treated sample is slightly weaker in terms of C–O bonds, and this may be due to the replacement of -OH to form P-O-C bonds by covalent interactions in cellulose [57]. The absorption peak is seen at 1662 cm^−1^, indicating the presence of N–H vibrations [58,59]. The side shift at the absorption peak is 1662 cm^−1^ and 1272 cm^−1^ corresponds to changes in the structure of the P-O and P-O-C groups [45]. The small peaks observed at 800 and 850 cm^−1^ may be the result of various salts [60]. Based on the results of this FTIR analysis, it can be confirmed that the FR treatment with the DM-3072N coating material was successfully impregnated into the ramie fabric.

The cellulose component of natural fibers generally does not exhibit flame-retardant properties and fire protection mechanisms [61]. The results of FR treatment show that a fabric made from natural ramie fiber can withstand fire due to the binding process and the presence of mineral salts in the FR formula.

#### 3.5.2. NMR

**Figure 3:**
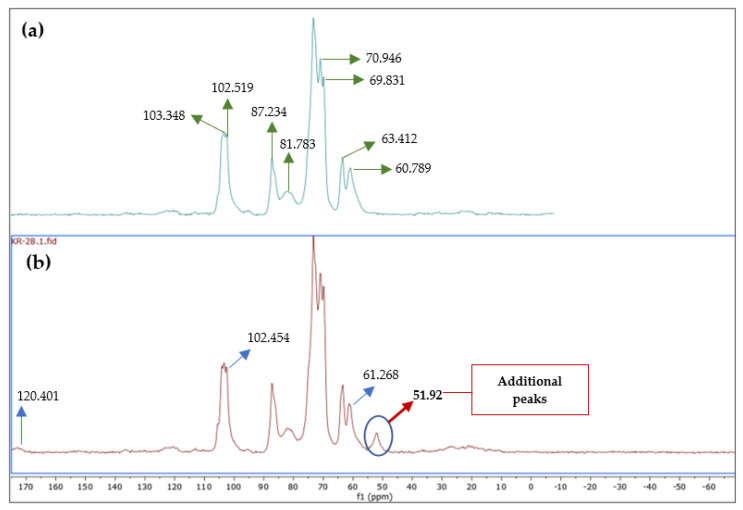
NMR analysis of ramie fabric (**a**) before treatment, and (**b**) with FR treatment.

**Figure 4:**
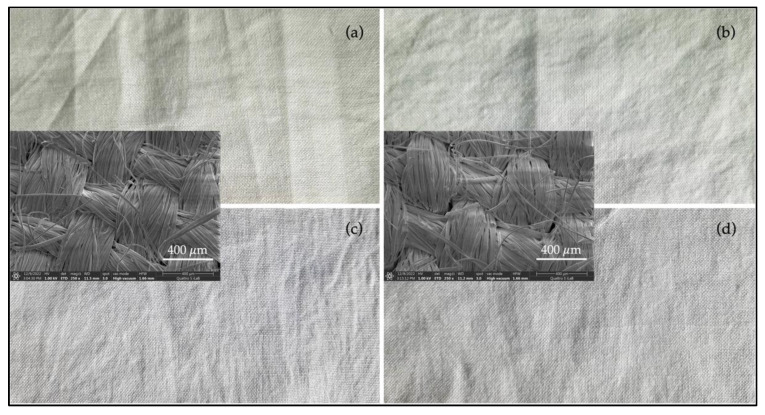
Optical microscope image: (**a**,**c**) KR-T01 ramie fabric without FR treatment, (**b**,**d**) ramie fabric with FR treatment (top image of fabric condition before washing and bottom image of fabric condition after washing).

The ^13^C NMR (101 MHz, CDCl_3_) test results confirm the presence of an additional peak in the ramie fabric after treatment. Based on the results, NMR analysis of the FR fabric after treatment shows the presence of chemical shift/additional peaks at δ 51.92 ppm (Figure 3b). The peak shift indicates the possible presence of CH_2_-N or CH-N which may have contributions in the fire-retardant effect of the ramie fabric (Figure 4). Further research using a ^31^P NMR test is needed to characterize the presence of the main flame-retardant element, phosphorus, in the treated fabric.

### 3.6. Morphological Characterization

The optical microscope images shown in Figure 4 revealed that ramie fabrics retained their fabric texture after treatment. Furthermore, FR-coated samples exhibited smoother surfaces compared to uncoated fabrics, as the active FR filled the gaps in the woven fabric structure. The weight of the ramie fabric before and after 50% FR treatment was 4.38 g and 4.88 g, respectively. With increasing concentrations of FR, the treated ramie fabric acquires weight gain and shows improved flame-retardancy behavior, which is indicated by the increased LOI and decreased char length [62].

The change in fabric size after washing also did not have a significant effect on the woven fabric. After washing, the woven fabric exhibited a shrinkage (reduction in size) of 3.21%. The ramie fabric that has undergone processing is in accordance with the standard testing value for the change in fabric size after washing, which is ±3% (ISO 6330). The results of this research showed that woven ramie fabric, formed into threads woven perpendicularly, maintains stability and resists deformation during the treatment process.

Testing with a scanning electron microscope (SEM) showed that the control ramie fabric exhibited a tightly woven structure with a dense arrangement of fibers. The fabric demonstrates a cross-weaving technique characterized by regular grooves and a dense configuration of warp and weft threads. The warp count comprises two strands of intersecting threads, while the weft count consists of a single strand. The untreated ramie fabric displays a relatively smooth surface structure without significant alterations.

## 4. Conclusions

This discovery reveals a lamination method utilizing non-toxic organophosphorus material, offering a practical, streamlined process that eliminates the need for layering lamination on ramie fabric. The inclusion of an FR agent content ranging from 40 to 50% enhances the flame-retardant properties of ramie fabric. The application of this resin demonstrates remarkable flammability characteristics, as evidenced by the vertical flame test, yielding a char length of 15–30 mm. These findings signify a heightened level of safety, particularly in containing fire spread on ramie fabric and extinguishing fires in potentially hazardous conditions. The simplicity of this process makes it a practical method for potentially transforming the characteristics of ramie fabric, which could be applied to various types of cellulose-based materials. The research findings reveal the promising development of flame-retardant ramie fabric, showcasing optimal flame-retardant properties with versatile application across industrial sectors. These include security industries necessitating protection against fire risks, such as flame-retardant attire for military personnel, firefighters, and workers in the oil and gas industry. Moreover, flame-retardant ramie fabric can also find utility in fire blankets and furniture materials like those for chairs, carpets, lampshades, curtain, and wall panels. These applications play a pivotal role in impeding fire spread and extending response time during emergencies.

## Figures and Tables

**Table 1:** Characterization of woven ramie fabric (KR-T01).

No.	Characterization	Woven Fabric	Standards Test
1.	Weave Type	Plain	SNI 0458:2013; SNI ISO 7211-1:2010
2.	Thread Number (tex)	25.26	SNI ISO 7211-5:2010
3.	Strand Yarn (strands/inch)	Warp: 52 Weft: 57	SNI ISO 7211-2:2010
4.	Fabric Thickness (mm)	0.28	SNI ISO 5084:2010
5.	Fabric Weight (g/m)	186.97	SNI ISO 3801:2010
6.	Fabric Width (cm)	159.64	SNI ISO 22198:2010
7.	Change in Size After Washing (%)	−3.21	SNI ISO 6330-2015
8.	Fabric Tensile Strength/2.5 cm (N)	Warp: 249.76 Weft: 207.38	SNI 0276-2009
9.	Fabric Tear Strength (n)	Warp: 55.52 Weft: 37.08	SNI 0521-2008

**Table 2:** The evaluation of combustion was conducted based on predefined criteria.

Criteria Conditions	Classification		
	B1	B2	B3
Char length (cm)	≤15	≤20	No special treatment
After flame time (s)	≤5	≤15	
After glow time (s)	≤15	≤30	

Notes: Char length: when measuring the fire resistance of textiles, the distance from the edge of the fabric, which is directly exposed to fire to the furthest point of visible fabric damage after the specified tearing force has been applied. After flame time: the length of time that a material continues to burn after cessation (natural or induced) of the material’s combustion. Afterglow time: the incandescent time continues after removal of the ignition source and/or cessation of ignition.

**Table 4:** Characterization of woven ramie fabric based on tensile strength.

No.	Material Fabric	Tensile Strength (MPa)
1.	KR-T01—non-FR (untreated)	56.69 ± 5.84
3.	KR-T01—50% FR	40.60 ± 3.32

**Table 5:** Limiting oxygen index (LOI) value for fabrics of different types and with different treatments.

No.	Material (Trademark)	Treatment	LOI (%)	Reference
1.	Ramie fabric	Untreated	22	this work
2.	Ramie fabric	Finished flamatic 50%	29	this work
3.	Cotton fabric	Spptms	28.5	[48]
4.	Cotton fabric	Coated T/C blend	27.1	[51]
5.	Cotton fabric	Finished	27.4	[52]
6.	Wool fabric	Bio-based phytic acid	23.6–27	[49]

## Data Availability

Data are contained within the article.

## Abbreviations

FR—flame retardant; CA—citric acid; SHP—Sodium Hypophosphite; H_2_O_2_—Hydrogen peroxide; NaOH—Sodium hydroxide; Resin—flammable organic substrate, produced to provide and preserve structure, stability, and viscosity.
